# Influenza A Virus Impairs Control of *Mycobacterium tuberculosis* Coinfection Through a Type I Interferon Receptor–Dependent Pathway

**DOI:** 10.1093/infdis/jit424

**Published:** 2013-08-09

**Authors:** Paul S. Redford, Katrin D. Mayer-Barber, Finlay W. McNab, Evangelos Stavropoulos, Andreas Wack, Alan Sher, Anne O'Garra

**Affiliations:** 1 Division of Immunoregulation, Medical Research Council National Institute for Medical Research, The Ridgeway, Mill Hill, London; 2 Division of Medicine, NHLI, Imperial College, London, W2 1PG, United Kingdom; 3 Immunobiology Section, Laboratory of Parasitic Diseases, National Institute of Allergy and Infectious Diseases, National Institutes of Health, Bethesda, Maryland

**Keywords:** *Mycobacterium tuberculosis*, tuberculosis, influenza, Interferon, type I IFN, co-infection

## Abstract

Influenza followed by severe acute bacterial pneumonia is a major cause of mortality worldwide. Several mechanisms account for this enhanced susceptibility, including increased production of type I interferon (IFN). In individuals infected with *Mycobacterium tuberculosis*, the influence of acute viral infections on tuberculosis progression is unclear. We show that prior exposure of mice to influenza A virus, followed by *M. tuberculosis* infection, leads to enhanced mycobacterial growth and decreased survival*.* Following *M. tuberculosis*/influenza virus coinfection, mycobacterial growth is enhanced by a type I IFN signaling pathway. Our findings highlight the detrimental influence influenza virus infection can have before or during *M. tuberculosis* infection.

Tuberculosis, caused by the bacterium *Mycobacterium tuberculosis*, and influenza, caused by influenza virus, together remain 2 of the greatest threats to global health. In the healthcare setting, the adverse effects of an influenza pandemic will initially be felt by patients with chronic respiratory conditions, including those with active tuberculosis [1]. Epidemiological and clinical data indicate that the majority of patients who die of influenza have severe bacterial pneumonia, the majority of cases of which are caused by acute infection with *Streptococcus pneumonia*, *Staphylococcus aureus*, and *Haemophilus influenzae* [2]. Experimental animal models also indicate that influenza A virus (IAV) infection significantly enhances susceptibility to secondary infection with these bacterial species, culminating in increased bacterial loads and reduced survival in coinfected animals [2–6]. Several mechanisms have been proposed to account for the increased susceptibility to secondary bacterial infection following IAV infection, including increased levels of type I interferon (IFN) [5]; desensitization to Toll-like receptor ligands, leading to defective myeloid cell recruitment [4]; impaired natural killer cell responses [6]; and production of antiinflammatory factors, including interleukin 10 [3].

Whether there is an interaction between influenza virus and more chronic bacterial infections, such as those due to *M. tuberculosis,* is less well understood, with limited clinical, epidemiological, and experimental data. Mathematical modeling, particularly of epidemiological data from influenza pandemics, point to an association between *M. tuberculosis* and IAV coinfection and increased mortality [7]. Only 1 study, published in 1947, has experimentally addressed the question of whether IAV increases susceptibility to tuberculosis in mice [8]. However, this study was limited by an inability to quantify bacterial load and by the use of a nonphysiological intravenous route of *M. tuberculosis* infection, and it only reported an increased number of *M. tuberculosis* lesions in the lungs.

The induction of type I IFN, which may occur during influenza virus infection, might be acting detrimentally during tuberculosis, which led us to reexamine the effects of IAV on bacterial control during pulmonary *M. tuberculosis* infection and whether this was type I IFN dependent. We found that IAV infection of mice before or during *M. tuberculosis* infection, enhanced susceptibility to tuberculosis. Furthermore, we reveal a detrimental role for type I IFN signaling in the IAV-mediated increase in the levels of mycobacteria in the lungs of *M. tuberculosis*/IAV coinfected mice.

## MATERIALS AND METHODS

### Mice

Female C57Bl/6 (wild type; WT) and C57Bl/6 × *Ifnar1*^−/−^ mice (here denoted “IFNαβR^−/−^”), were bred and housed under specific-pathogen-free conditions at the United Kingdom Medical Research Council (MRC) National Institute for Medical Research (NIMR) or were purchased from Taconic Farms (Germantown, NY). All animals were bred and maintained for experiments in accordance with either United Kingdom Home Office regulations or at an Association for Assessment and Accreditation of Laboratory Animal care International–accredited animal facility at the National Institute of Allergy and Infectious Diseases (NIAID), National Institutes of Health, and were used in accordance with an animal study proposal approved by the NIAID Animal Care and Use Committee (Bethesda, MD).

### In vivo *M. tuberculosis* and IAV Infections

*M. tuberculosis* experiments were performed under biosafety level 3 conditions. *M. tuberculosis* H37Rv was prepared by growing H37Rv bacilli to mid-log phase in Middlebrook 7H9 broth supplemented with OADC (Difco), quantified on 7H11 agar plates, and stored in aliquots at −80°C. For aerogenic infections, a Glas-Col or 3-jet Collison nebulizer unit (BGI, USA) was used, delivering approximately 50–150 colony-forming units (CFU) to the lungs. IAV subtype A/Puerto Rico/8/34(H1N1) (hereafter, PR8) was a kind gift from Drs Kanta Subbarao and Yuk-Fai Lau. IAV subtypes X31 (a subtype H3N2 reassortant with a PR8 backbone) and A/California/04/09(H1N1) (hereafter, “Cal/09”; kind gifts from Dr J. Skehel, MRC NIMR). All IAV subtypes were grown in the allantoic cavity of 9–10-day-embryonated specific-pathogen-free hen's eggs, sterile filtered, and free of bacterial or mycoplasma contamination. IAV was delivered intransally under isoflurane anesthesia at the dose and infection schedules outlined in the figure legends.

### Tissue Harvest

Lungs were aseptically removed from mice and homogenized by passing through a 70-μm or 100-µm nylon sieve with the piston of a 2-mL syringe. Cell suspensions were serially diluted onto 7H11 agar plates supplemented with OADC (10%), and after 18–21 days at 37°C, visible CFU were counted and the lung mycobacterial loads calculated.

### Histopathologic Analysis

Lungs were fixed by inflating the tissues with 4% formaldehyde, sectioned, and stained with hematoxylin-eosin. Scoring of inflammation was done in a blinded fashion by a pathologist.

### Statistical Analysis

All data were analyzed as indicated in the figure legends, with differences being considered significant when *P* < .05.

## RESULTS

**Figure 1.**
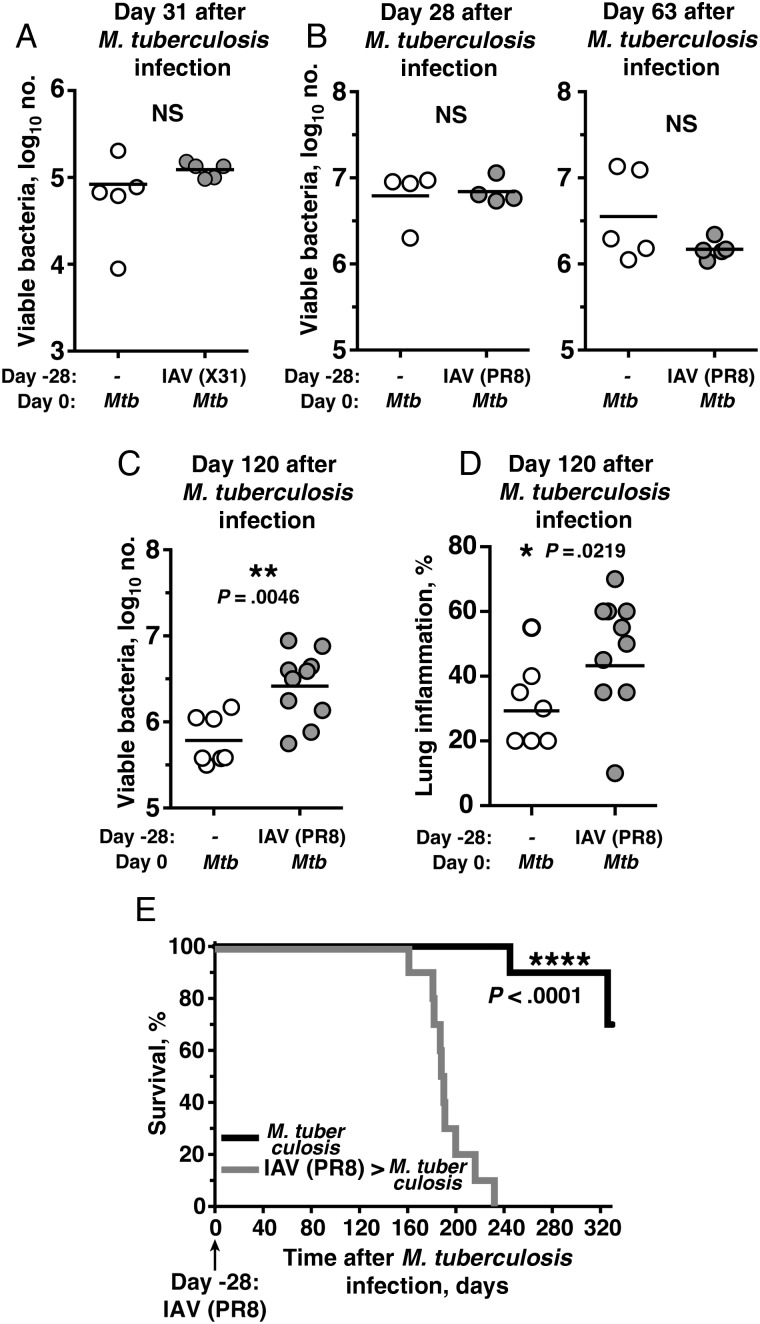
Prior exposure to influenza A virus (IAV) predisposes mice to enhanced susceptibility to subsequent *Mycobacterium tuberculosis* infection. Mice were first infected intranasally with IAV subtypes X31 (median tissue culture infective dose [TCID_50_] of 1 × 10^4^; *A*) or PR8 (TCID_50_ of 100; *B*–*E*) or were mock infected with phosphate-buffered saline. A total of 28 days later, mice were challenged via aerosol with *M. tuberculosis*, and the numbers of viable mycobacteria in the lungs were determined at the time points indicated. The extent of inflammation in the lungs (*D*) and survival (*E*) following *M. tuberculosis* infection was also monitored in animals (8–10 mice per group) that had or had not been previously infected with IAV subtype PR8. Results shown are representative of at least 2 independent experiments, with individual data points depicting individual mice. Statistically significant differences in the numbers of viable mycobacteria in the lungs between groups were assessed using the Mann–Whitney *U* test, with the resulting *P* values indicated. The differences in survival between control and coinfected mice were determined by the log-rank test, with the resulting *P* value indicated.

We sought to determine the influence of IAV infection before and during acute *M. tuberculosis* infection by looking at its effect on mycobacterial control in the lungs. To investigate the effect of IAV infection before *M. tuberculosis* challenge, mice were sequentially infected via the intranasal route with IAV subtype X31 or PR8 and were allowed to rest for 28 days to allow viral clearance before infection with *M. tuberculosis*. Following aerogenic *M. tuberculosis* infection, mice that had previously been infected with either IAV subtype X31 (Figure 1*A*) or subtype PR8 (Figure 1*B*) showed no significant differences in lung mycobacterial loads as compared to mice infected with *M. tuberculosis* alone at the time points indicated. In addition, flow cytometric analysis of CD4^+^ and CD8^+^ T-cell populations recognizing the mycobacterial specific antigens ESAT-6 and TB10 on day 28 after *M. tuberculosis* infection showed no significant differences between mice previously infected with IAV 4 weeks before *M. tuberculosis,* compared with those infected with *M. tuberculosis* alone (data not shown). However, despite the initial lack of an effect of prior IAV infection on susceptibility to tuberculosis (Figure 1*A* and 1*B*), significant increases in lung mycobacterial load (Figure 1*C*) and inflammation severity (Figure 1*D)* were observed on day 120 after *M. tuberculosis* infection in mice previously infected with IAV, compared with findings for controls infected with *M. tuberculosis* alone. Moreover, the observed increase in mycobacterial load more than 120 days after *M. tuberculosis* infection resulted in a significant decrease in survival (Figure 1*E*).

How IAV infection impairs the subsequent control of *M. tuberculosis* infection during the chronic stages of tuberculosis in our study is not known. However, the strongly delayed effects of previous IAV exposure on *M. tuberculosis* control and the significant increase in lung mycobacterial levels observed on day 120 after *M. tuberculosis* infection suggest that IAV induces subtle long-term modifications to the lung microenvironment, which may be more important at later stages of *M. tuberculosis* infection and, thus, ultimately responsible for the reduced late-stage survival seen among mice infected with IAV followed by *M. tuberculosis*.

**Figure 2.**
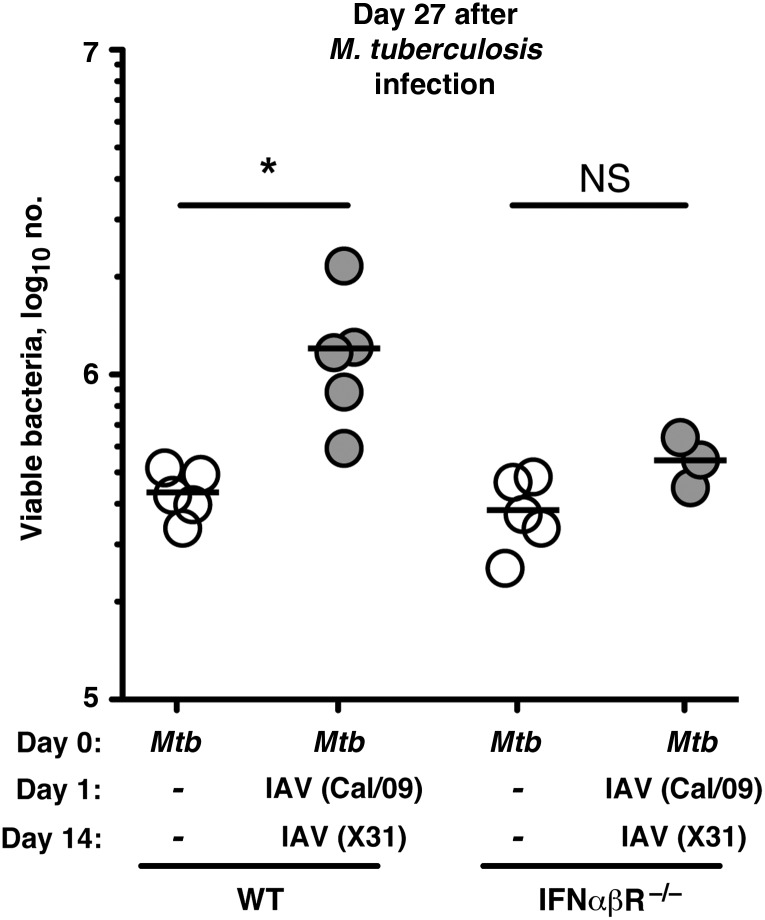
Concurrent influenza A virus (IAV) challenge impairs *Mycobacterium tuberculosis* control by a mechanism dependent on type I interferon (IFN) signaling. Mice were first infected via aerosol with *M. tuberculosis* and then were intranasally challenged with IAV (or were mock infected with phosphate-buffered saline) on day 1 (Cal/09; [TCID_50_] of 1 × 10^4^) and day 14 (X31; TCID_50_ of 2.4 × 10^4^) after *M. tuberculosis* infection. Mice were killed on day 27 after *M. tuberculosis* infection, and the numbers of viable mycobacteria in the lungs were determined. Results shown are representative of 2 independent experiments, with individual data points depicting individual mice. Statistical comparisons were performed using 1-way analysis of variance with a Bonferroni multiple comparison posttest. *P < .05. IFNαβR ^–/–^, C57Bl/6 × *Ifnar1*^−/−^ mice; Abbreviations: NS, nonsignificant; WT, wild-type mice.

We have shown that prior infection of mice with IAV 28 days before aerosolized *M. tuberculosis* challenge has a detrimental influence on mycobacterial control and on survival. However, since a significant proportion of the underlying mechanisms of IAV-bacterial interactions have been studied in the context of acute coinfection [9], we sought to address the influence of *M. tuberculosis*/IAV coinfection on mycobacterial control in the lungs. Mice were infected via aerosol with *M. tuberculosis* and then challenged sequentially via the intranasal route with 2 different IAV subtypes, one (Cal/09) on day 1 and another (X31) on day 14 after *M. tuberculosis* infection. Mycobacterial loads were then determined in the lungs on day 27 after *M. tuberculosis* infection. This IAV infection schedule was chosen because it is known that IAV subtypes H1N1 and H3N2 are actively circulating in human populations and because more than one IAV subtype may be in seasonal circulation, giving rise to individuals potentially being exposed to more than one IAV subtype [10]. Following *M. tuberculosis*/IAV coinfection of WT mice, we observed a significant increase in the lung bacterial loads, compared with loads in WT controls infected with *M. tuberculosis* alone (Figure 2). The enhanced lung mycobacterial loads in WT animals was not observed when mice were coinfected with *M. tuberculosis* and only 1 IAV subtype (Cal/09) (Supplementary Figure 1). Based on the published effects of IAV on acute bacterial infections such as *S. pneumonia*, several mechanisms could be responsible for this observed increase in bacterial levels in *M. tuberculosis*/IAV-coinfected mice, including the production of type I IFN. The induction of type I IFN during intracellular bacterial infections, such as those due to *Listeria monocytogenes*, is associated with suppression of the innate immune response and loss of resistance to infection [9]. With these studies in mind, we investigated the role of type I IFN in promoting mycobacterial growth in concurrent *M. tuberculosis*/IAV-coinfected mice. Following *M. tuberculosis*/IAV coinfection, we showed that the significant increase in lung mycobacterial growth seen in coinfected WT mice was dependent on type I IFN signaling, as IFNαβR^−/−^ mice coinfected with *M. tuberculosis*/IAV had mycobacterial loads comparable to those in control IFNαβR^−/−^ mice infected with *M. tuberculosis* alone (Figure 2).

## DISCUSSION

The induction of type I IFN during primary infection with bacterial pathogens has been associated with a detrimental disease outcome [5, 9]. More recently, it has been shown that IAV infection significantly enhances host susceptibility to secondary bacterial infection, culminating in increased bacterial loads and reduced survival in coinfected animals [2–6]. We show here for the first time that IAV infection of mice before *M. tuberculosis* infection significantly impairs long-term mycobacterial control in the lungs, leading to a decrease in host survival. In addition, coinfection of mice with *M. tuberculosis* and IAV enhances mycobacterial growth in the lungs through a pathway dependent on type I IFN signaling. The findings we present here highlight a novel mechanism for the exacerbated disease observed in the epidemiological studies of individuals coinfected with *M. tuberculosis* and IAV [7]. Furthermore, our data also complement previous studies that used animal models, which highlighted that type I IFN has a negative influence on the immune response during *M. tuberculosis* infection [11]. Such studies have shown that treatment of mice with IFNαβ [12] or that exogenous induction of type I IFN by Poly-ICLC treatment can exacerbate disease via regulation of the interleukin 1 pathway [13]. In line with this, patients with active tuberculosis, compared with individuals with latent infection and healthy controls, have both a type I IFN and type II IFN–inducible blood transcriptional signature that correlates with the radiographic extent of disease and disappears on successful treatment, further suggesting a negative role for type I IFN in the pathogenesis of tuberculosis [14].

The potential mechanisms underlying the detrimental effect of type I IFN during *M. tuberculosis* infection as observed in our study could include the downregulation of the IFN-γ receptor [15], of importance since macrophage activation by IFN-γ is essential for eradicating intracellular bacterial infections. On the other hand, type I IFNs induced by IAV may mediate the deregulation of the chemokine balance required for the optimal recruitment of myeloid cells needed to control secondary bacterial infections [5]. Finally, antiinflammatory factors such as interleukin 10 [3], which itself can be regulated by type I IFN, may be induced following IAV infection and can block protective immune responses during *M. tuberculosis* infection [11]. Therefore, the production of immunoregulatory factors can compromise the hosts' ability to control primary and secondary bacterial infections, inadvertently promoting disease.

In summary, given the ever-increasing global prevalence of tuberculosis, our findings highlight the detrimental influence that IAV infection can have either before or during *M. tuberculosis* infection by significantly enhancing the hosts' susceptibility to tuberculosis. Our findings are applicable to a pandemic influenza setting but are also germane to seasonal influenza, in which, under certain scenarios, the outcome of infection may vary according to the status of the population under study, which could be influenced by other clinical manifestations, immigration to new geographical locations, changes in the local pathogen prevalence, and host genetic factors. These findings are also in line with data from epidemiological studies of individuals coinfected with *M. tuberculosis*/IAV during influenza pandemics. More pertinently, we reveal a novel detrimental role for type I IFN signaling in the IAV-mediated enhancement of tuberculosis in coinfected mice.

## Supplementary Data

Supplementary materials are available at *The Journal of Infectious Diseases* online (http://jid.oxfordjournals.org/). Supplementary materials consist of data provided by the author that are published to benefit the reader. The posted materials are not copyedited. The contents of all supplementary data are the sole responsibility of the authors. Questions or messages regarding errors should be addressed to the author.

Supplementary Data
