# Supplementary material for: Influenza A Virus Impairs Control of Mycobacterium tuberculosis Coinfection Through a Type I Interferon Receptor–Dependent Pathway
Source: J Infect Dis. 2013 Aug 9;209(2):270–4. doi: 10.1093/infdis/jit424 (PMC3873785; doi:10.1093/infdis/jit424)
Supplement: Supplementary Data [file supp_209_2_270__index.html]

Influenza A virus impairs control of Mycobacterium tuberculosis co-infection through a Type I Interferon receptor dependent pathway — Influenza A Virus Impairs Control of Mycobacterium tuberculosis Coinfection Through a Type I Interferon Receptor–Dependent Pathway — Influenza A Virus Impairs Control of Mycobacterium tuberculosis Coinfection Through a Type I Interferon Receptor–Dependent Pathway — Supplementary Data 

# Influenza A Virus Impairs Control of *Mycobacterium tuberculosis* Coinfection Through a Type I Interferon Receptor–Dependent Pathway

## Supplementary Data

Supplementary Data

**Files in this Data Supplement:**

- Supplementary Data - Docx file
