# Supplementary material for: Influenza A Virus Impairs Control of Mycobacterium tuberculosis Coinfection Through a Type I Interferon Receptor–Dependent Pathway
Source: J Infect Dis. 2013 Aug 9;209(2):270–4. doi: 10.1093/infdis/jit424 (PMC3873785; doi:10.1093/infdis/jit424)

**Supplementary Figure 1: *M. tuberculosis* / IAV co-infection of mice using only a single IAV subtype (Cal/09; H1N1) at day 1 post *M. tuberculosis* has no significant effect on lung bacterial loads.** Mice were first infected via aerosol with *M. tuberculosis (Mtb),* and then intranasally challenged with IAV (or mock infected with PBS) at day 1 (Cal/09; 1x10^4^ TCID50) post *M. tuberculosis* infection. Mice were killed at day 28-post *M. tuberculosis* infection and viable mycobacteria were determined in the lungs. Statistical comparisons were performed using an unpaired Students *t* test (NS = Non-Significant), with individual data points depicting individual mice.


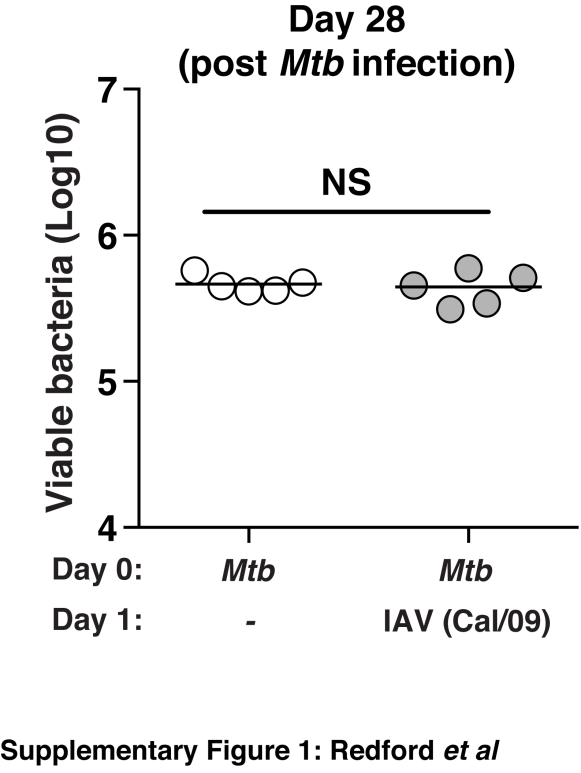

Supplement: Supplementary Data [file supp_jit424_jit424supp.docx]
